# Immune-Mediated Inner Ear Disease Associated with Type 1 Autoimmune Hepatitis: A Challenging Coexistence

**DOI:** 10.31138/mjr.33.3.349

**Published:** 2022-09-30

**Authors:** Athina Zarachi, Eleftherios Pelechas, Alkistis Tsikou, Georgios N. Kalambokis, Aikaterini Zioga, Katerina E. Panteli, Iro Rapti, Sempastian Filippas-Ntekouan, Ioannis Kastanioudakis, Fotini B. Karassa

**Affiliations:** 1 Department of Otorhinolaryngology, Faculty of Medicine, School of Health Sciences, University of Ioannina, Ioannina, Greece,; 2 Department of Rheumatology, Faculty of Medicine, School of Health Sciences, University of Ioannina, Ioannina, Greece,; 3 1^st^ Department of Internal Medicine, Faculty of Medicine, School of Health Sciences, University of Ioannina, Ioannina, Greece,; 4 Department of Pathology, Faculty of Medicine, School of Health Sciences, University of Ioannina, Ioannina, Greece

**Keywords:** immune-mediated inner ear disease, type 1 autoimmune hepatitis

## Abstract

Autoimmune hepatitis (AIH) is characterized by elevated serum transaminase, increased immunoglobulin G levels, presence of autoantibodies, and hepatocellular damage. Coexistence with other autoimmune diseases has been reported in almost half of patients with AIH. Here, we report a 60-year-old man who developed rapidly progressive, bilateral, asymmetrical, and asynchronous sensorineural hearing loss that was consistent with immune-mediated inner ear disease (IMIED). This devastating presentation evolved as a late manifestation in the context of a six-month systemic illness that had previously resulted in type 1 AIH. A biochemical remission with normalization of aminotransferases achieved within two months after the initiation of corticosteroids with azathioprine. Further, an acceptable response has also been achieved at the patient regarding the right ear-hearing impairment; though, treatment could not reverse the substantial decrement in hearing capability of the left ear. To our knowledge, this is the first case report of the concurrent development of type 1 AIH and IMIED.

## INTRODUCTION

Autoimmune hepatitis (AIH) is a chronic inflammatory liver disease that is char-acterised by elevated transaminase levels, increased immunoglobulin G levels, autoantibodies, and histological features of interface hepatitis.^1–4^ AIH is a relative rare disorder with its incidence rates in adults to range between 0.67 to 2 cases per 100,000 person-years^3^ while its prevalence vary from 16 to 18 cases per 100,000 inhabitants in Europe.^5^ Yet, other autoimmune diseases co-aggregate in up to 44% of patients with AIH, particularly in those aged ≥60 years.^3^

Immune-mediated inner disease (IMIED) is an uncommon cause of bilateral sensorineural hearing loss (SNHL), reflecting an organ-specific inflammatory process confined to the inner ear.^6–10^ It is frequently associated with some primary forms of vasculitis, mostly Cogan syndrome (CS), granulomatosis with polyangiitis (GPA) and giant cell arteritis (GCA).^10^ IMIED also occurs in the context of certain systemic autoimmune disorders such as systemic lupus erythematosus (SLE) and sarcoidosis.^6–10^ The incidence of IMIED has been estimated to be less than 5 cases per 100,000 per year.^7^

Here, we present the first case, to our knowledge, of the sequential presentation of type 1 AIH and IMIED.

## CASE DESCRIPTION

**Figure 1.**
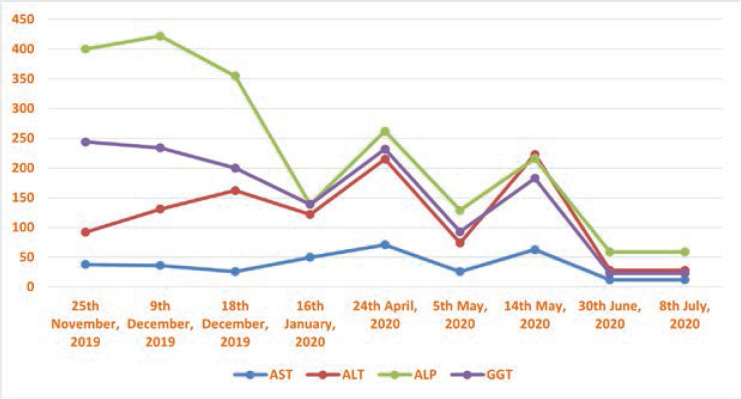
Trends of liver function tests (IU/L) during patient’s sequential evaluations.

A 60-year-old man was initially hospitalised in the dermatological department of our hospital for low-grade fever, slightly raised erythematous lesions and swelling in both ankles, particularly in the right one. His medical history only included congenital right hip dislocation. The patient did not receive any medications. He consumed alcohol occasionally and not above recommended limits. Measurements of C-reactive protein (CRP) and of erythrocyte sedimentation rate (ESR) showed raised values. Elevated liver enzymes were also found (**Table 1**, **Figure 1**). Other laboratory tests were within normal limits. Tests for hepatitis A, B and C were negative as also rheumatoid factor and anti-cyclic citrullinated peptide antibody. Iron, ferritin and ceruloplasmin levels were within normal limits. A chest radiograph did not reveal hilar adenopathy or pulmonary infiltrates and levels of angiotensin converting enzyme were normal. Ultrasound imaging of the right ankle only detected tenosynovitis of the tibialis posterior muscle while duplex ultrasonography did not show findings of vein thrombosis. The patient received a diagnosis of possible erysipelas and empirical treatment with cefuroxime was administered. The symptoms abated substantially.

**Table 1.** Main laboratory findings during patient’s evaluations.

**Variable**	**Reference Range**	November 25^th^, 2019 *	December 14^th^, 2019 **	April 24^th^, 2020 ***	**May 14^th^, 2020 †**	**July 8^th^, 2021 ¶**
Haematocrit (%)	41–53	40.6	34.6	46.8	42.3	49.9
Haemoglobin (g/dl)	13.5–17.5	13.2	11.8	15.6	14.3	16.5
White-cell count (per μl)	4500–11,000	8900	12,510	15,240	8510	6310
C-reactive protein (mg/L)	< 6	156	208	89	22	1
Erythrocyte sedimentation rate (mm/h)	0–30	69	68	81	58	2
Aspartate aminotransferase (AST) (IU/L)	10–35	38	36	71	63	20
Alanine aminotransferase (ALT) (IU/L)	10–35	92	181	215	223	21
Gamma-glutamyltransferase (GGT) (IU/L)	10–52	244	215	232	183	16
Alkaline phosphatase (ALP) (IU/L)	30–125	400	257	262	216	67
IgG concentration (mg/dl)	751–1560	1400	1360	1500	1300	NA

NA: not available

* during his hospitalisation in the dermatological department;

** during his 1^st^ hospitalization in the internal medicine department;

*** during his 2^nd^ hospitalization in the internal medicine department;

† during his hospitalization in department of otorhinolaryngology;

¶ during his more recent evaluation at the rheumatology clinic.

**Figure 2.**
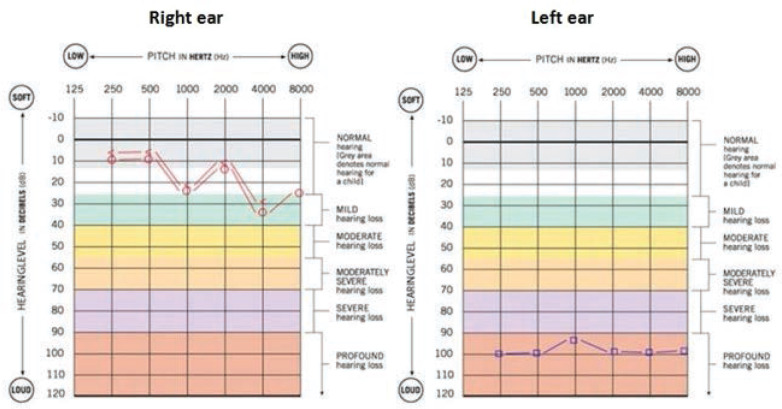
First audiogram, consistent with profound hearing loss of the left ear (90–100dB).

Two weeks later he was admitted to the internal medicine department (second admission) because fever recurred. The patient also reported weight loss and low back pain. The clinical examination revealed mild tenderness on direct palpation of the left sacroiliac joint whereas no source of sepsis was found. Laboratory testing showed normochromic, normocytic anaemia, leucocytosis, as well as markedly elevated ESR, CRP, and liver enzymes (**Table 1**). Treatment with ciprofloxacin was started. Repeated blood cultures, urine culture, microbial serology, and echocardiography were all negative. Magnetic resonance imaging (MRI) was contraindicated due to a metallic foreign body after surgery. Hence, three phase scintigraphy and computed tomography (CT) scan were undertaken that showed possible sacroiliitis in the left sacroiliac joint probably due to excess mechanical load owing to congenital right hip dislocation and associated pelvic dysplasia. Based on these findings, the diagnosis of sacroiliac septic arthritis was deemed more likely and teicoplanin was added. CT of the chest and the abdomen were performed revealing mild fibrous elements in the upper lobes of lungs. No bile duct stenosis or other lesions were observed. The liver was homogenous without focal lesions. Mantoux test was negative. Eight days later fever persisted. Liver enzymes (**Figure 1**) and acute-phase reactants remained elevated, and thus, the initial treatment was discontinued. Piperacillin-tazobactam and linezolid were started. Two weeks later the symptoms gradually improved, and the patient was discharged home. Oral ciprofloxacin with clindamycin were administered for two months after his discharge, yet ESR and liver enzymes remained elevated. Blood tests were positive for antinuclear antibodies (ANA) at a titre 1/160 with a speckled pattern. Anti-double-stranded DNA (anti-dsDNA), anti-Sm, anti-Ro/SSA, anti-La/SSB, anti-RNP, anti-ribosomal P, and antiphospholipid antibodies were all negative. A repeat CT showed no abnormal findings in the left sacroiliac joint. Four months later, the patient developed sudden hearing loss and tinnitus with a sense of aural fullness in the left ear. Oral corticosteroids (cs) were empirically administered by an otolaryngologist for five days. The symptoms did not improve, and therefore, the patient was readmitted to the internal medicine department (third admission). Neurologic examination and otoscopic findings were normal. For the assessment of hearing loss, the 512-Hz tuning fork was used. The Rinne test was positive (air conduction > bone conduction) while in the Weber test, sound lateralized to the right ear suggesting SNHL in the left ear. The audiogram showed a normal hearing threshold in the right ear and a hearing threshold of 90 decibel (dB) compatible with deafness in the left ear (**Figure 2**). Laboratory tests remained abnormal (**Table 1**, **Figure 1**). Serum IgG concentration was close to the upper limit of normal (1500 mg/dl, reference range: 751–1560 mg/dl-Table). A liver biopsy was performed given the considerable and persistent elevation of transaminases as well as of enzymes associated with cholestasis (**Table 1**, **Figure 1**); yet, intravenous (iv) cs had already been started with an initial dose of 250 mg methylprednisolone daily for four days and a gradual tapering thereafter. The regimen was immediately given as first-line treatment for the profound SNHL in the left ear following the recommendations of the otolaryngologists. Smooth muscle antibodies (SMA) and perinuclear antineutrophil cytoplasmic antibodies (p-ANCA) were detected at a titre 1/40 and 1/80, respectively whereas antimitochondrial antibodies (AMA) were negative. Symptoms stabilized and the patient was discharged home while waiting the results of the liver biopsy.

**Figure 3.**
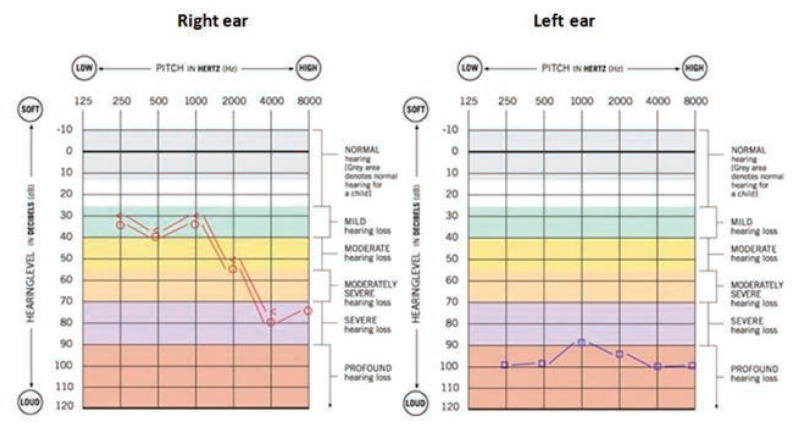
Second audiogram, consistent with moderately severe hearing loss of the right ear particularly on high frequency sounds.

**Figure 4.**
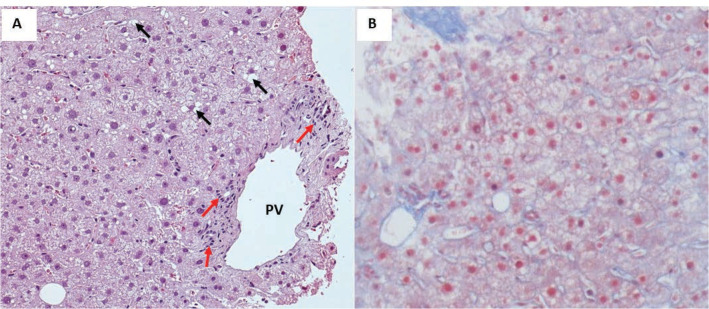
Pathological examination of liver specimens. Few lymphocytes in a portal tract and steatosis (**a**) Black arrows: fat vacuoles; Red arrows: lymphocytes; PV: portal vein (haematoxylin and eosin; magnification x100). No fibrosis was detected in the liver biopsy examination (**b**) (Masson trichrome; magnification x100).

Three days later he was admitted to the department of otorhinolaryngology (fourth admission) because he presented hearing loss in the right ear as well associated with tinnitus. He also reported remarkable difficulty understanding conversations conducted amid background noise. Findings with the 512-Hz tuning fork and the audiogram confirmed the symptoms described and revealed a moderate SNHL in the right ear (**Figure 3**). Since MRI with gadolinium that is considered the diagnostic procedure of choice for identifying retrocochlear lesions causing SNHL was contraindicated, he underwent the auditory brainstem responses. The waveforms were recorded at 80 decibels for the left ear and at 70 decibels for the right ear. The findings were consistent with asymmetric, bilateral SNHL. Values of CRP and liver tests remained high (Table and Figure 1). The results of brain CT were virtually normal. High-resolution CT of the temporal bones did not reveal lytic findings or erosions; bilateral cervical lymph nodes with a diameter of ≤ 10 mm and benign characteristics were found. The devastating nature of bilateral SNHL required timely and aggressive immunosuppression with cs, and therefore, he received three intratympanic dexamethasone injections in both ears. The liver biopsy revealed lymphocytic inflammatory infiltration of the portal tract and mild findings of non-alcoholic fatty liver disease (NAFLD) (**Figure 4A**) without hepatic fibrosis (**Figure 4B**) or histopathological demonstration of granuloma. The results from a specialized center confirmed positivity for p-ANCA at a titre 1/80 (positive titre>1/20). SMA were positive at a titre 1/160 (positive titre>1/40) and directed against filamentous actin (anti-F-actin SMA). AMA, liver-kidney microsome antibodies (anti-LKM), anti-soluble liver antigens/liver pancreas antibodies (anti-SLA/LP), and antibodies to liver cytosol antigen type 1 (anti-LC1) were all negative. Indirect immunofluorescence (IIF) on rodent multi-organ (kidney-liver-stomach) substrates was used for the detection of liver-related autoantibodies, apart from anti-SLA where immunoblotting was the method of testing. ELISA was also used that confirmed the negative result for IgG anti-SLA/LP. Quantitative values of serum IgG and IgA antimitochondrial M2 antibody (IgG-M2; IgA-M2) levels obtained with ELISA were under the cut-off of the method. AMA have also been tested for by immunoblotting and were negative. Non-detection for anti-LC1 was confirmed by immunoblot as well. Anti-gp210 and anti-sp100 of IgG isotype were negative; both autoantibodies were tested by ELISA. ANA (detected by IIF testing on HEp-2 cells) were positive at the same titre and with speckled fluorescence pattern as previously; yet anti-myeloperoxidase (MPO) antibodies were negative. Levels of C4 were relatively low during this additional evaluation (14 mg/dl, reference range: 16–38 mg/dl).

## DIFFERENTIAL DIAGNOSIS

The diagnosis of definite autoimmune hepatitis (AIH) of type 1 was made based on the revised original scoring system of the International Autoimmune Hepatitis Group (IAIHG)^1^ that appears to have better diagnostic performance in individuals who have few or atypical disease features, but other scoring systems could also be used with similar specificity.^11,12^ The patient did have elevated aminotransferase levels (aspartate [AST] aminotransferase and alanine aminotransferase [ALT]), and a cholestatic liver enzyme profile (alkaline phosphatase [ALP] and gamma-glutamyltransferase [GGT]) with an ALP/ALT ratio < 1.5^1^ in most laboratory investigations and as the immune-mediated inflammatory liver disease evolved (**Table 1**, **Figure 1**); yet increased serum IgG concentration was not detected. Elevated IgG is considered an important marker but normal levels do not preclude the diagnosis of AIH.^5,13^ In fact, IgG levels are normal in up to 15% of patients with this clinical entity^4,5,14^ whereas increased levels of IgG are not disease-specific findings.^15^ The autoantibody profile (ANA, SMA [anti-F-actin], and p-ANCA) reflected the characteristic serological repertoire for AIH of type 1^3–5,13^ whereas bilirubin was normal, and AMA were negative using multiple assays. Interestingly, detection of multiple autoantibodies, as those found at the patient, and especially the combination of ANA and SMA has specificity 99%^4,16^, a positive predictive value of 97%, and a diagnostic accuracy of 74% for AIH^4^. Hence, this combination may distinguish this clinical entity from other chronic liver diseases that have different immune-mediated mechanisms.^16^ In addition, the liver histology revealed portal lymphocytic inflammatory infiltration that was compatible with the diagnosis^1,3–5,12,13^ but without lymphoplasmacytic cells extending into the lobule, emperipolesis, or rosettes. Emperipolesis and hepatocyte rosetting are histologic findings associated with more severe necroinflammatory and fibrotic changes^17^ that may point to the diagnosis of AIH,^1,3–5,12,13^ albeit non-specific features.^17,18^ Further, cs had already been started to the patient before the liver biopsy and probably modified the burden of the portal inflammatory infiltrates.^3^ Mild histological features of steatosis were also found in liver specimens; in fact, findings of NAFLD appear in 17%–30% of patients with AIH^3^ particularly in males.^19^ Instead, findings that depict chronic biliary disease or overlap syndrome such as bile duct injury, portal granulomas, florid bile duct lesions or the concentric, so-called onion-skin periductal fibrosis which is the classic histologic hallmark for primary sclerosing cholangitis (PSC)^3^ were not demonstrated. Moreover, the patient did not fulfil the “Paris criteria”; these criteria may identify individuals with overlapping features of AIH and primary biliary cholangitis (PBC).^3,20^ AMA are being detected in more than 90% of PBC patients. Nonetheless, if the patient were to have AMA-negative PBC or a variant syndrome^3,22^, then diagnostic performance would be improved by testing for PBC-specific nuclear autoantibodies.^22^ PBC-defining ANA are characterized by multiple nuclear dots or rim-like/membranous pattern by using IIF on HEp2 cells^22^; yet such fluorescence patterns have not been demonstrated at the patient. Moreover, anti-gp210 and anti-sp100 antibodies that result in the specific fluorescence patterns^22^, were also negative. He had no history or symptoms indicative of ulcerative colitis which is frequently present in patients with AIH–PSC overlap syndrome^3,4,21^ while CT of the abdomen did not reveal bile duct lesions. Even if the patient had persistent cholestatic laboratory abnormalities, the histological features that were not consistent with PSC and the non-demonstration of bile duct lesions on CT also argues against small-duct or large-duct PSC respectively.^3,21^ The patient also developed the devastating manifestation of rapidly progressive SNHL that became bilateral within three weeks. The clinical presentation was asynchronous and asymmetric. The process was consistent with IMIED^6–10^ since time course is a principal criterion for distinguishing other disorders from this entity and common causes of inner ear dysfunction had been excluded based on imaging and other diagnostic studies.^6–10,23^ Teicoplanin that has been administered to the patient is ototoxic only in the presence of renal failure^24^; yet his renal function was normal. Piperacillintazobactam has also been associated with tinnitus and potentially with sudden hearing loss.^25^ Nevertheless, these symptoms tend to develop promptly after drug administration^24^; the patient presented with SNHL and tinnitus four months after receiving these antibiotics. In fact, SNHL developed as a late manifestation in the context of a systemic illness that unfolded during a six-month course and had previously resulted in type 1 AIH. Bacterial, viral, fungal, and parasitic pathogens including Mycobacterium tuberculosis have all been implicated for liver function test abnormalities.^26^ Nevertheless, infectious aetiologies had been meticulously ruled out through the history including travel and exposures, physical examination, blood cultures, appropriate microbiologic and imaging studies as well as liver biopsy findings during the patient’s initial hospitalizations. Further, if the six-month multi-organ systemic illness was to be due to infectious causes it would be expected to follow a more aggressive clinical course. Consequently, the differential diagnosis based on the findings of IMIED, systemic symptoms, the protracted disease course and the laboratory tests included CS, ANCA-associated vasculitis, polyarteritis nodosa (PAN), GCA, SLE as well as other immune-mediated diseases, and in particular, sarcoidosis, Behcet’s disease, and IgG4-related disease (RD).^27–42^

### Cogan syndrome

It is an immune-mediated disease that primarily affects young adults. The hallmark of CS is the presence of ocular and audiovestibular symptoms.^10,32,33^ These findings may be accompanied by evidence of systemic vasculitis.^10,32^ The most common ocular symptoms include interstitial keratitis, uveitis, and episcleritis. Inner ear disease is manifested by SNHL and vestibular dysfunction.^10,32,33^ Abnormal vestibular function is found in 90% of patients with CS^10^ who typically present with abrupt onset of tinnitus and vertigo. These symptoms often subside but are followed by progressive SNHL which may be both unilateral and bilateral^33^, primarily affecting middle and high frequencies with fluctuations.^10^ Inner ear involvement in CS develops with a prevalence between 31% and 45%; yet in 50%–60%, bilateral deafness and tinnitus develops over time.^10^ Our patient did not have symptoms suggestive of ocular inflammation. Further, he did not present the typical pattern of vestibular involvement that has been described in CS while vasculitic manifestations reflecting arteritis, particularly aortitis, aortic aneurysms, and aortic and mitral valvulitis^32^ were absent. Hepatitis is also not commonly seen with this disease.^43^ Hence, the patient’s age at disease onset, the absence of hallmark manifestations of CS and the findings that were consistent with AIH of type 1 make this diagnosis unlikely.

### ANCA-associated vasculitis

This group of systemic vasculitides comprises GPA (formerly Wegener’s granulomatosis), microscopic polyangiitis (MPA) and eosinophilic granulomatosis polyangiitis (EGPA; formerly Churg-Strauss syndrome).^32^ While this group of disorders is often associated with ear symptoms and hearing impairment may be one of the presenting manifestations in GPA and a common finding in EGPA, involvement of the middle ear predominates leading to a higher frequency of conductive hearing loss than SNHL; asymmetrical SNHL appears to be a relatively infrequent feature of ANCA-associated vasculitis.^10^ The patient presented with bilateral SNHL as a late manifestation during the evolution of the systemic illness. In addition, rapidly progressive glomerulonephritis is a typical feature of ANCA-associated vasculitis^32^ that develops in up to 80% of patients with GPA or MPA^34,35^; however, the patient did not have any laboratory findings suggestive of renal involvement. Another hallmark of this group of vasculitis is the involvement of upper and lower respiratory tract.^32,34^ The patient did not report any symptoms that would indicate respiratory tract involvement and he did not also have imaging findings that characterize ANCA-associated vasculitis.^32,34^ Further, the lack of other essential features^32,34,35^, and the non-detection of anti-MPO which in combination with p-ANCA pattern on immunofluorescence testing have a high positive predictive value for ANCA-associated vasculitis, most commonly MPA^32,34^, rule out these disorders.

### Polyarteritis nodosa

PAN is a necrotizing arteritis of small and medium-sized muscular arteries. This vasculitis variant frequently causes constitutional symptoms, while it has a predilection for certain organs, particularly the skin, peripheral nerves, gastrointestinal tract, and kidneys primarily causing renovascular hypertension and renal infarctions.^36^ Hepatic artery involvement is common in PAN leading to moderate elevation in aminotransferase levels.^36^ The disease preferably affects men aged around 50 years. SNHL often occurs in patients with PAN; it is typically bilateral and symmetrical, with sudden onset caused by fibrosis with ossification in the scala tympani of the basal turn.^8^ Numerous pathological findings have been reported and includes vasculitis of the labyrinthine artery with ischemic necrosis of cochlear and vestibular labyrinths, cochlear neural degeneration, fibrous tissue proliferation in the inner ear, as well as atrophy of the stria vascularis.^10^ The patient presented constitutional symptoms, anaemia of chronic disease, neutrophil leucocytosis, persistently elevated inflammatory markers, arthralgias, and SNHL. Nonetheless, if he were to have PAN, it would be expected to see nodular skin lesions, ulcers or livedo reticularis, clinical findings suggestive of mononeuritis multiplex and the elevation in aminotransferase levels should reflect the degree of hepatic artery involvement and the resulting ischemic changes. In addition, the patient’s normal renal function, the autoantibody profile, and the liver histology that was compatible with the diagnosis of type 1 AIH make PAN unlikely.

### Giant cell arteritis

GCA is a granulomatous arteritis usually affecting the aorta with a predilection for the branches of the carotid and vertebral arteries. It is characterised by the frequent involvement of the temporal artery.^32,44^ Constitutional symptoms, headache, visual symptoms, jaw claudication, scalp tenderness, and abnormally appearing temporal artery are the typical manifestations and findings of GCA while it is often associated with polymyalgia rheumatica.^44^ Audiovestibular symptoms that may develop in GCA include SNHL frequently presenting as sudden or rapidly progressive unilateral or bilateral hearing loss, vertigo, and otalgia.^8^ Biochemical abnormalities of liver function, more often elevated ALP occur in nearly 50% of patients with this form of systemic vasculitis, while AST and ALT can also be mildly raised in up to 40% of them.^45^ The aetiology of abnormal liver enzymes remains unclear; yet, it has been postulated that elevated ALP results from cholestasis secondary to ischemic injury and rarely from granulomatous arteritis.^45^ Even though the patient presented with constitutional symptoms and rapidly progressive, bilateral SNHL that may appear in GCA, he also had characteristic laboratory findings, the autoantibody repertoire and liver histologic features that were consistent with type 1 AIH. The diagnosis of this vasculitic syndrome is also implausible based on lack of other prominent disease manifestations and clinical findings.

### Systemic lupus erythematosus

Constitutional symptoms, arthralgias of ankles, lymph-adenopathy, positive ANA, relatively diminished levels of C4, SNHL^8^ and type 1 AIH42 might be consistent with the diagnosis of lupus, albeit with a less typical presentation. SNHL is the most common audiovestibular symptom which occurs in 6%–70% of lupus patients; hearing loss may be bilateral or unilateral but it typically affects high frequencies.^8,10^ Vasculitis triggered by immune complex deposition is the most important cause of cochlear and vestibular damage in lupus patients.^8,10^ Antiphospholipid antibodies have also been implicated in the pathogenesis of inner ear dysfunction in SLE patients and mostly in the occurrence of SNHL that seems to be related to microthrombus formation in the labyrinthine vasculature^8^; yet the negative tests argue against their involvement in the occurrence of SNHL in this patient. AIH has a prevalence ranging from 1.7% to 19.4% in SLE.^42^ SLE–AIH overlapping is defined by the presence of fulfilling ACR classification criteria for SLE in a patient who also meets IAIHG criteria for AIH.^42^ Still, it is imperative to distinguish lupus hepatitis from AIH. The most common feature of lupus hepatitis is subclinical liver disease without markedly elevated levels of liver enzymes.^42^ In addition, anti-ribosomal P are useful serological markers to differentiate among these two entities; these autoantibodies are positive in almost half of patients with lupus hepatitis but are rarely detected in AIH.^42^ On the other hand, anti-F-actin SMA characterize AIH^3–5,13,18^ and are nearly absent in lupus hepatitis.^42^ Liver biopsy is, still, crucial to make the correct diagnosis^42^; lupus hepatitis most commonly includes lobular infiltrates with few lymphocytes^42^ while AIH is primarily characterized by interface hepatitis with emperipolesis, rosettes, and centrilobular necrosis depending on the severity of the inflammatory response.^3–5,13^ The patient had persistently elevated liver enzymes, negative anti-ribosomal P testing, the typical serological repertoire of type 1 AIH, and histopathology that was compatible with this disorder. Hence, although some clinical and laboratory features could be consistent with SLE, the fact that this patient is male, and the absence of other major disease manifestations makes its diagnosis unlikely. The autoantibody profile was not also in favour of lupus as anti-dsDNA and anti-Sm which are highly specific for this disease, were negative. Furthermore, he cannot be classified as SLE according to either the 1997 updated ACR^46^ or the 2019 EULAR-ACR new classification criteria that appear to have excellent diagnostic accuracy for the disease.^47^

### Sarcoidosis

It is a systemic disease due to noncaseating epithelioid granulomatous inflammation in affected organs.^38,39^ A high incidence of audiovestibular manifestations has been reported that occur in the context of neurosarcoidosis.^8^ Hearing loss, either unilateral or bilateral, may appear in nearly all patients with sarcoidosis.^8^ Tinnitus also develops in about 61% of patients with this disease.^8^ Yet, unlike this patient, severe vestibular symptoms including vertigo, dizziness, and paroxysmal positional vertigo with abnormal vestibular functioning tests appear in approximately 90% of sarcoidosis cases.^8^ Furthermore, the absence of pulmonary involvement with the characteristic chest imaging abnormalities which appear in 70–90% of patients with sarcoidosis^38,39^ and, more importantly, the lack of noncaseating granulomatous inflammation in liver specimens^48^ exclude sarcoidosis.

### Behçet’s disease

It is characterized by the presence of recurrent oral and genital ulcers, ocular inflammation, and skin lesions.^40^ Small vessel vasculitis, arteritis, arterial aneurysms, venous thrombosis, and arterial thromboangiitis may also occur.^32^ It is principally a disease of young adults.^40^ Hearing loss is a frequent manifestation in this disorder.^8,10,41^ It is primarily bilateral and usually affects high frequencies; several studies have reported SNHL following cochlear impairment ranging from 12% to 80% in Behcet’s disease patients while approximately half of them also have abnormal vestibular function.^8,10,41^ Even though SNHL often occur in Behcet’s disease, the absence of other defining manifestations of this disorder in the patient, as also his age when the symptoms first developed, make this diagnosis improbable in this case.

### IgG4-related disease

IgG4-RD is a multi-organ immune-mediated disease that imitates various malignant, infectious, and inflammatory disorders.^29,30^ Hepatic involvement^49^ and particularly laboratory features of cholestasis^28^ as well as SNHL could be indicative of IgG4-RD. Since pancreato-hepatobiliary disease is the potential clinical phenotype^30^ for this patient, then morphological changes of affected organs would be expected to be found. In particular, diffuse pancreas enlargement that usually encompasses more than two-thirds of the pancreas or a tumour-like mass are common imaging findings,^27,29,30^ as type 1 autoimmune pancreatitis is associated with IgG4-related sclerosing cholangitis in almost 90% of cases.^28^ In addition, it would be anticipated stenosis of the bile duct since the type of biliary involvement that is highly consistent with IgG4-related sclerosing cholangitis affects the proximal biliary tract.^28^ The usual clinical presentation of autoimmune pancreatitis is obstructive jaundice induced by concomitant IgG4-related sclerosing cholangitis, secondary diabetes mellitus that occurs in about half of cases, and malabsorption due to exocrine insufficiency.^29,30^ The patient did not have such manifestations whereas recurrent fever that was a dominant symptom of his initial presentation is uncommon in IgG4-RD.^29^ Further, the nearly diagnostic CT features of autoimmune pancreatitis that include diffuse organ enlargement with delayed enhancement and a capsule-like low-density rim^29,30^ have not been demonstrated in the patient. Narrowed lesions of the bile duct have not also been detected. It is also remarkable that the liver biopsy did not reveal typical histologic findings of IgG4-associated AIH, or IgG4-related sclerosing cholangitis such as storiform fibrosis, obliterative phlebitis, significant accumulation of plasmacytes in the liver, lymphoplasmacytic infiltrates surrounding the bile ducts, or tissue eosinophilia.^29,49^ On the other hand, SNHL is a rare manifestation of IgG4-RD^29^ where pachymeningitis, mass forming lesions or erosions of the temporal bone are frequently described^31^; yet, such imaging findings were not found at the patient. The absence of other organ involvement, such as IgG4-related dacryoadenitis/sialadenitis, retroperitoneal fibrosis, or renal lesions that may coexist in about 26% of patients with IgG4-related sclerosing cholangitis^28^ also makes the disease unlikely. Interestingly, in IgG4-RD, ESR can be elevated to a moderate degree whereas CRP is usually normal apart from certain clinical manifestations, namely retroperitoneal and aortic involvement, in which a slight increase can be observed.^30^ These findings are in sharply contrast with the marked elevation of these acute phase reactants (**Table 1**) that was persistently found during the patient’s disease course. Peripheral blood eosinophilia and increased serum IgE levels that occur in almost 30% of cases^30^ were not found as well. Lastly, according to recently developed classification criteria,^27^ IgG4-RD could not also be regarded as a diagnostic option for this patient.

## CLINICAL COURSE

**Figure 5.**
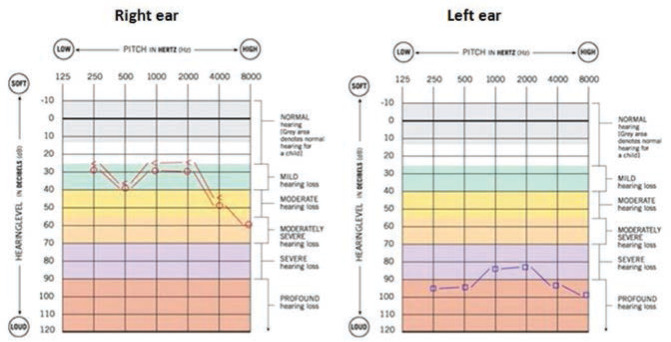
Third audiogram. Improvement of the right ear-hearing acuity by approximately 20 dB after 3 intratympanic dexamethasone injections followed by 3-day intravenous methylprednisolone at a dose of 500 mg/d. Oral prednisolone with azathioprine were subsequently started. This audiogram was done approximately 2 months after the initiation of oral treatment.

With the final diagnosis of type 1 AIH and IMIED, oral prednisolone in combination with azathioprine (AZA) was decided to start and after the evaluation by a rheumatologist. This combined therapy is a common treatment option^6–10^ for both immune-mediated diseases and was given according to current guidelines for the management of AIH.^3,5,13^ Intravenous methylprednisolone (500 mg daily for 3 days) preceded the administration of oral treatment. Since approximately 3 gr of iv methylprednisolone were administered overall to the patient at the early stage of IMIED and considering the long-term side-effects of cs, it was decided to start oral prednisolone at a dose 30 mg/d in combination with azathioprine that was gradually increased to 125 mg daily. This decision was primarily based on both the clinically acceptable audiologic improvement of the patient and the current guidelines for the management of AIH.^3^ Values of CRP and ESR gradually reverted to normal. A biochemical remission with normalization of aminotransferases had been achieved within two months that permitted a gradual taper of prednisolone by approximately 2.5 mg every four weeks. The normalization of both ALP and GGT (**Table 1**, **Figure 1**) following the initiation of oral treatment was consistent with the histological findings reflecting the absence of an additional liver disease component of cholestasis and especially argues against the diagnosis of AIH–PSC variant syndrome.^3^ The regimen also halted the inflammatory process regarding the right ear-hearing impairment since a hearing gain of 20 dB in pure tone average (mean of 500, 1000mean of 500, 2000, 4000 Hz) was achieved. A plateau of recovery was reached approximately 2 months after the initiation of oral treatment as the last audiogram showed (**Figure 5**). Still, immunosuppressive treatment could not reverse the significant decrement in hearing capability of the left ear. The patient currently presents with an acceptable level of speech discrimination in a quiet environment with no other clinical manifestations and with sustained normal serum levels of liver enzymes. However, communications in the presence of background noise are quite difficult; therefore, hearing aids or a cochlear implant^50^ are appropriate options for the patient over the next months to improve his quality of life and to diminish the risk of late-life dementia.^51^

## DISCUSSION

Other autoimmune diseases are present in a significant proportion of patients with AIH^3,4^ whereas IMIED is a common feature of some forms of primary vasculitis or certain systemic disorders.^8,10^ Nevertheless, to our knowledge, this is the first described case of the co-presentation of these two immune-mediated entities.

The clinical presentation of AIH in adults is extremely heterogeneous, ranging from an asymptomatic state to acute liver failure.^3,4,18^ Since there is no single diagnostic test for AIH, the disease diagnosis in the patient was supported by the elevated aminotransaminase levels, his autoantibody profile, the exclusion of other liver diseases that may resemble AIH, and also by the compatible histological findings.^3,4,18^ In 1993, a diagnostic scoring system for AIH was compiled by the IAIHG group,^52^ then revised in 1999,^1^ and subsequently simplified in 2008.^12^ These scoring systems were principally developed to ensure the comparability of study populations in clinical trials rather than diagnosing AIH in individual patients. Yet, the revised original scoring system and mostly the simplified score are recommended as useful tools for the disease diagnosis in daily clinical practice.^3,5.13^ Unfortunately, in a substantial proportion of cases, the clinical presentation is less typical.^3,4,18^ Scoring systems can aid in establishing the diagnosis of AIH in such challenging cases, as in the patient who did not have increased IgG and some histological characteristics. The revised original scoring system^1^ is preferable in such patients^2^ and it is also recommended as a helpful tool in diagnosing difficult AIH cases.^13^ The simplified scoring system^12^ is more likely to result in the exclusion of cases with unusual features.^3,5^ Therefore, we used the revised original scoring system^1^ for establishing the diagnosis of AIH in the patient, as it appears to have higher sensitivity (100% vs 95%) than the simplified score^12^ in cases who lack classical features.^2^ These scoring systems are not fully interchangeable^2^; hence, in specific clinical situations such as the coexistence of other immune-mediated diseases and in cases where normal serum IgG levels or high-titre autoantibodies are found, as in this patient’s presentation, the revised original scoring system^1^ has better diagnostic performance than the simplified scoring system.^2,3.5,11^ According to the revised original scoring system^1^, the pre-treatment score for the patient was of 16 points and the posttreatment of 18 points; these aggregate scores were both consistent with definite diagnosis of AIH. Interestingly, a pre-treatment score of 15 points, indicative of definite AIH has a sensitivity of 95%, a specificity of 97%, and a diagnostic accuracy of 94%.^53^ According to the simplified scoring system,^12^ the score for the patient was 5 and not diagnostic for the disease; yet, cases with suspected AIH not reaching a diagnostic score result with this scoring system should be reassessed using the revised original criteria.^3,18^ More importantly, in daily clinical practice, any scoring system has only to be considered as an aid to AIH diagnosis and the relevant criteria should be used in common with clinical judgement.^3,5,13^

IMIED usually develops in middle-aged individuals; SNHL in this inflammatory disorder is typically bilateral, albeit the left and right ear may be affected asymmetrically and asynchronously.^6–10^ Disturbances of balance can occur as well since the inner ear also mediates vestibular function.^6–10^ The time course of IMIED is relatively rapid, and hence, prompt initiation of cs along with a cytotoxic agent is crucial to restore hearing loss.^6–10,54^ Our case poses, however, the dilemma about the intensity of immunosuppression after irreversible organ damage has occurred. A clinically acceptable audiologic improvement has been achieved at the patient, with the iv administration of methylprednisolone as also the intratympanic dexamethasone and subsequently with oral prednisolone and AZA.^54^ This improvement concerned the right ear-hearing impairment given the hearing gain of 20 dB in pure tone average. Treatment could not, however, reverse the substantial decrement in hearing capability of the left ear. Therefore, a reasonable approach is essential to immunosuppressive treatment selection that will be able to adequately control disease manifestations but without placing patients at risk for undesirable toxicities. T cell-mediated autoimmunity might be the pathogenic link for the concurrent development of type 1 AIH and IMIED.^3,6–9^ CD4^+^ T cells are considered the crucial pathogenic drivers in AIH.^3,4^ TNF-producing CD4^+^ T cells are aberrantly activated Th1 cells and these subsets are likely to be involved in the pathogenesis of AIH.^3,4,55^ Interestingly, animal models provide evidence that IMIED is also a T cell-mediated organ-specific autoimmune disorder where activated CD4^+^ T cells with a Th1 phenotype have been implicated in the induction of this manifestation.^8,56^ A break in self-tolerance to several autoantigens that triggers dysregulated immunological responses against different cellular targets has also been involved in the pathogenesis of both diseases.^3,4,6–9,15,56,57^ Autoantibodies are one of the typical features of AIH.^3–5,13,15,18^ Regarding IMIED, injury to the stria vascularis and hair cell death are associated with immune complex deposition.^8,57^ Other vessel lumen-obstructive events involving microthrombus formation and recruitment of inflammatory infiltrates at the arterial wall which result to vascular insults also shape the pattern of inner ear involvement in IMIED.^8,57^

Hair cell stereocilia structure is implicated in mechano-sensing and depends on actin filaments.^58–61^ Cochlear hair cells initiate the process of hearing by converting mechanical deflections of their stereocilia bundles into neural action potentials.^58–61^ Remarkably, mature cochlear hair bundles encompass tightly controlled numbers of actin-filled stereocilia; the distal tip compartment is composed of F-actin and is renewed at a faster rate than the actin core. F-actin in the distal tip seems to regulate stereocilia tip dynamics near to the site of mechano-electric transduction.^58–61^ Hence, SMA with specificity for F-actin which characterize type 1 AIH,^3–5,13,15,18^ and detected in the patient, might have disrupted F-actin-based structures of stereocilia bundles impairing hearing transduction^58–61^ and have induced SNHL. This process could be proposed as an additional pathogenic mechanism that have been triggered by immune responses to certain autoantigens, specifically F-actin. This putative mechanism might well point to a link for the sequential development of type 1 AIH and IMIED in the patient.

## CONCLUSION

In conclusion, we describe the first case of IMIED presenting as a late manifestation in the context of a systemic illness that had previously caused type 1 AIH. Awareness is needed to keep in the differential diagnosis and other than previously reported autoimmune diseases as possible associations of IMIED when clinically relevant symptoms or laboratory findings appear.
